# Eye, Ear, Nose and Throat

**Published:** 1893-04

**Authors:** 


### EYE, EAR, NOSE AND THROAT.

EDITED BY DR. ARTHUR G. HOBBS, ASSISTED BY
DR. GEORGE BROWN.

### HYPERTROPHY OF TONSILS.

Dr. Moure, in *Le Bulletin Medical*, distinguishes three types of hypertrophied tonsils. 1. The pedunculated. 2. The encysted. 3. The psuedo-hypertrophic.

In the first form he advises removal with the bistoury, or galvanic snare, and advises anesthetization with the bromide of ethyl.

In the second form he advises the use of pastes of iodine and chromic acid, with electrolysis, and ignipuncture or thermo-cautery, or the galvano-cautery.

In the third form he advises the cautery and local applications in the crypts of each ; also the insertion of a blunt hook and tearing out the crypts bodily. The base to be cauterized with a probe dipped in the following solution:

℞. Trichloracetic acid, gr. i½.

Iodine, gtt. iv.

Iodide potash, gr. viii.

Glycerine, ℨii¼.

M. Distilled water, ℨiiss.

Sir Morrell McKenzie recommends the application of perchloride of iron, tannin or alum. For the cases where the glands were really hypertrophied he used London paste or excision· Dr. Solis Cohen recommends electrolysis, a slow and tedious way of removing the gland.

Sajous says that alum and tannin seem to be the only astringents that are capable of any good results in these cases. He says active treatment is always indicated when the tonsils are large enough to occasion complications or interfere with proper breathing.

He uses the bistoury tonsillotome, wire snare and galvano-cautery.

Hobbs says: "When the hypertrophy is due to thickening of the connecting tissue nothing short of excision can ever reduce the gland. He uses his bistoury tonsillotome, which consists of a knife curved on the flat and a double hook, for raising the tonsil out of its bed. It can be easily loosened by a slight rotation of the handle in case of gagging. He uses this instrument in 75 per cent. of his cases. He does not in any case ever enucleate a tonsil, but attempts to excise the hypertrophied mass only down to the level. In short he removes just enough of the enlargement to relieve the throat of the sensation of a foreign body.

Knight highly praises the galvano-cautery in patients with a hemorrhagic tendency.

Dr. Th. Goureau says that it can be summed up as follows: painting, excision and actual cautery. He advises excision with Massoneuve's Amygdalotome.

The treatment after excision is merely astringent and disinfectant gargles, with nitrate of silver applications to all granulations that appear unhealthy.

The oozing which may follow excision may be controlled by the gallo-tannic acid gargle, composed of six drams of tannic acid and two of gallic acid in an ounce of water.

---

Otitis Media is discussed from its surgical aspects by Dr. Jack, in the *Boston Medical and Surgical Journal.* He concludes: 1. The removal of the drum membrane and ossicles is attended with little annoyance to the patient, proof of which is suffient to warrant the performance of the operation as the only means of cure in many cases. 2. The operation often produces marked improvement of the hearing. 3. Satisfactory results may be expected toward the relief of tinnitus and vertigo. 4. The results of the operation seem to be permanent."—*Med. Record.*

